# Supplementary material for: Are carbon emissions trading and green financial instruments synergistic? -Comprehensive quantitative research based on content analysis
Source: PLoS One. 2024 Mar 7;19(3):e0298601. doi: 10.1371/journal.pone.0298601 (PMC10919601; doi:10.1371/journal.pone.0298601)
Supplement: S1 Data — (ZIP) [file pone.0298601.s001.zip › Data/地级市Z_F工作报告.zip!/╜¡╬≈/╞╝╧τ╩╨/└┤╫╘╣1⁄2╓┌║┼í╛┬φ┐╦╩2╛▌═°í┐_2015.docx]

各位代表：

现在，我代表市人民政府向大会作政府工作报告，请予审议，并请各位政协委员和其他列席人员提出意见和建议。

一、回顾2014年：在艰难转型中实现经济社会稳步发展

2014年是全面贯彻落实党的十八届三中全会精神和全面深化改革的第一年，也是转型发展极为困难的一年。在这个结构调整的阵痛期和增长速度的换挡期，在省委、省政府和市委的正确领导下，在市人大、市政协的监督支持下，全市着力稳增长、调结构、促改革、惠民生，经济社会发展平稳有序，基本完成了年初确定的主要目标任务。

(一)经济保持稳定增长。全年预计完成生产总值860亿元，增长8.5%；全社会固定资产投资908.3亿元，增长9.8%；规模以上工业增加值420亿元，增长9.5%；财政总收入117.06亿元，增长6.6%；社会消费品零售总额266.2亿元，增长12%；城镇居民人均可支配收入25899元，增长9%；农村居民人均可支配收入12750元，增长11%。全市存款余额683.92亿元，比年初净增60.50亿元；贷款余额458.21亿元，比年初净增77.38亿元；存贷比和新增存贷比分别达到67%和127.9%，中长期贷款增速居全省第三。着力推进项目建设，全年确定的124项重点项目累计完成投资830.16亿元。引进5000万元以上省外资金项目163个，实际进资335.52亿元。引进比克新能源客车、中材风电叶片等一批大项目，华能安源电厂、华美立家、赣湘国际物流港、江西宝安新材料等一大批项目顺利推进。

(二)产业结构逐步优化。产业经济协调发展，三次产业比为6.6：59.5：33.9。农业经济稳步发展，全年粮食作物播种面积125.5万亩，增长0.3%，粮油生产保持稳定。粮食收购创历史新高，为种粮农民增收1700万元。预计全市实现农业总产值88亿元，农业增加值57亿元，增长均为4%，全市拥有各类农业产业化组织2017个，市级以上龙头企业155家，新增34家。芦溪县现代农业示范区成功创建国家级现代农业示范区，农产品精深加工园建设扎实推进。“格林米特”杜仲猪肉成为南京青奥会唯一指定猪肉供应产品。“莲花老表”大米获得江西省首批农产品气候品质认证。工业发展稳中有进。五大传统产业预计完成增加值231.2亿元，占全市工业增加值的55%，增长9.5%，对全市工业增长贡献率达到50.2%。五大战略性新兴产业预计完成增加值81.7亿元，占全市工业增加值的19.4%，增长12.2%，对全市工业增长贡献率达到24.6%。工业陶瓷电瓷产业集群、粉末冶金产业集群入选我省首批20个省级工业示范产业集群，其中粉末冶金产业已有25家企业落户，8家企业投产，粉末冶金产业规划展示馆、粉末冶金产业孵化中心、综合性技术检测中心、科技创新创业中心全面封顶。积极实施科技创新战略，国家创新型试点城市建设取得明显成效。市本级连续8年获全国科技进步先进市称号。成功获批“国家火炬计划粉末冶金特色产业基地”和“国家电瓷高新技术产业化基地”。第三产业持续增长。大力实施旅游景区双5A战略，扎实推进旅游工作六大工程。武功山金顶整治初步完成，34栋违章建筑全部拆除，新山门、石鼓寺等基础设施建设进展较快，武功山温泉豪生度假酒店已开业运营，安源国家矿山公园项目基本完工，安源红领巾少儿基地建设顺利推进，武功山景区和安源景区产业集群入选省重点旅游产业集群。杨岐山景区和荷花博览园获评国家4A级旅游景区，孽龙洞景区成功改造提升，中国红鱼文化村被评为江西省4A级乡村旅游点。成功举办第七届武功山帐篷节、首届武功山全国大学生户外运动挑战赛和莲文化旅游节。全年实现旅游综合收入143.1亿元，增长39.2%。文化产业稳步发展，预计实现文化产业主营业务收入210亿元，增长10%。金融业稳步推进。萍乡小微企业信用体系试验区被列为江西唯一全国首批小微企业信用体系建设试验区，小微企业贷款余额达185.8亿元。萍乡农村商业银行、九江银行萍乡分行挂牌成立，交通银行萍乡市分行正式获国家银监会批准筹建。华维电瓷在“新三板”挂牌。政银企合作不断深化，市政府与10家省级银行机构签订了战略合作框架协议，3年拟贷款503亿元，在全省率先开展普惠金融活动。电子商务产业裂变式发展，电子商务交易总额122亿元，总量列全省第三，增幅273.84%，列全省第一。物流业蓬勃发展，上栗县永璟、强泰等物流集团竞相发展，达金物流被交通部、公安部共同指定为2014APEC会议燃放烟花产品唯一物流承运企业。

(三)新型城镇化和基础设施建设得到加强。坚持规划先行、城乡统筹，强化中心城区规划依法集中统一管理，编制了《萍乡市新型城镇化规划(2014—2020年)》。全年开工城镇化项目48个，完工17个，完成投资86.58亿元。全市城镇化率达到63.95%。加大了新农村建设力度，投入资金1.27亿元，完成308个村点建设和改造任务，农村生活环境不断改善。全面完成了高铁及地方配套工程，9月份高铁正式通车，衡茶吉铁路地方配套工程竣工，萍乡陶瓷产业基地和华能安源电厂的铁路专用线全面完成。高铁、高速、国省道完成投资43.2亿元，灾害恢复重建、大中修等项目完成投资5.45亿元。偿还道路建设贷款债务3.07亿元,分摊化解320国道萍乡段债务1.854亿元。公路建设落实计划项目里程747.8公里，项目资金1.2亿元；争取农村公路计划600公里，基本实现组组通水泥公路。萍洪高速、吉莲高速公路建设进展顺利，春节前将全面建成通车；昌栗高速上栗段建设进入高潮。萍高路竣工，320国道芦溪、湘东段改造提升工程顺利完成，通久路万新段扎实推进，萍福路路面破损修复完工。319国道城区段即将实现右幅单线通车，320国道城区段完成招投标。田中生态水库的路网框架建设成型，挖湖工程进入扫尾阶段。莲花(南岭)220KV输变电工程、芦溪县首座220KV变电站柳江变电站、萍乡北220KV牵引站线路工程、虎形山110KV输变电工程正式投运。天然气推广利用工作进展顺利。全市水利建设项目新增投资10.45亿元，其中争取上级补助资金7.4亿元，再创历史新高。节约集约用地扎实推进，顺利通过国家土地南京督察局专项检查。

(四)生态文明建设扎实推进。全年完成减排项目76个，完成污水管网建设、改造25.5公里。化学需氧量、氨氮排放量、二氧化硫、氮氧化物等指标实现下降，空气质量和水质有所好转。萍乡被列为全国水生态文明城市建设试点市。创建国家级生态乡镇15个、国家级生态村2个。大力推进封山育林，造林绿化完成省下达任务的124.4%。创建森林乡镇10个、森林村庄100个。市本级创建国家森林城市获得批复；芦溪县成功创建省级森林县城、园林县城、卫生县城；上栗县申报创省级森林城市已经获批；莲花县创省级森林城市通过省级验收；山口岩国家级湿地公园顺利通过国家预验收。推进了319、320、芦万武等5条富裕和谐秀美乡村示范带建设，综合环境进一步改善。

(五)民生事业成效显著。全面完成了省政府下达的民生工程指标。建立多层次、多类型的创业孵化基地，打造四级劳动就业创业公共服务平台，新增城镇就业31000人，新增农村劳动力转移就业22000人，城镇就业率达95.7%，零就业家庭就业安置率100%。所有县区全部纳入了新农保和城居保范围，全面实施了城镇职工基本医疗保险市级统筹和城镇居民基本医疗保险门诊统筹。矿区农贸市场、金陵大市场等农贸市场改造有序推进。全市保障性住房2800套全部开工建设；廉租住房租赁补贴完成13073户；棚户区改造完成9821户，农村危房改造完成5785户，中央下放煤矿棚户区改造项目开工15053户，建成8223户。山口岩水利枢纽工程全面竣工，全面实现了供水、防洪、发电、灌溉的功能。白源水厂建成投产，新增供水能力10万吨/天。投资近1亿元新建和改造城市输供水管网50公里，实现了白源、麻山和五陂下三个水厂的互连互通，互为备用水源，城市供水的水量、水压、水质、应急能力都有了根本性的保证。住房公积金管理安全规范。全面推进各项社会事业。全年教育资金投入23.1亿元，素质教育进一步提升，职业教育稳步推进，高考再创佳绩，教师队伍素质和管理得到提高。江西工业工程职业技术学院搬入新校区。上栗县被列为全省11个义务教育学校校长教师交流轮岗试点县之一。加强公共文化服务体系建设，文化事业进一步繁荣，话剧《老阿姨》广受好评，广播剧《本色》荣获中宣部“五个一工程奖”。安源大剧院开始运营。全市数字影院实现县区全覆盖。医药卫生体制改革不断深化，芦溪县县级公立医院和市二医院城市公立医院综合改革得到省政府肯定。全力开展食品药品安全专项整治，切实保障公众饮食用药安全。市体育中心改造工程全面完成。我市在第十四届省运会上金牌数、奖牌数、团体总分榜实现新突破，均比上届前进了3位。稳步推进单独二孩政策实施，低生育水平继续保持稳定。在全省实施妇女儿童发展纲要主要目标综合考评中，我市名列第一。市民宗局荣获全国宗教工作系统信息工作先进集体。政府应急管理工作取得新成绩，综合应急预救援队伍建设取得阶段性成果。强化了安全生产组织领导，构建了基层安全生产监管网络。建立了煤炭、烟花爆竹、非煤矿山和危险化学品四大高危行业安全综合监控平台，有力提升了全市本质安全水平。全市较大事故得到有效控制。安全生产监督管理工作获全国先进。信访工作取得“六个没有发生”的好成绩。成功应对了“5·25”历史罕见特大洪灾。社会保持和谐稳定，全市公众安全感和公安工作满意度均居全省第一。

(六)各项改革蹄疾步稳。在全省率先推行以“行政审批目录清单、市场准入负面清单、市场主体监管清单、审批流程再造和规范中介机构改革”为内容的“三单两制”改革，得到省政府主要领导的高度评价。市本级行政审批事项从原有的343项精简到114项 落霞小说。项目办理时限由574个工作日缩减到33个工作日。坚持“调”“控”同步推进政府机构改革，提升政府治理能力和服务水平。深入推进财政改革、工商注册制度改革、国资国企改革、教育领域综合改革、国有林场改革、集体林权制度配套改革等重点领域的改革，农村土地承包经营权确权登记颁证工作扎实推进，新城区和开发区成功合并。加快区域合作步伐，区域经济发展活力不断释放，赣湘开放合作试验区列入中央苏区振兴发展规划，并初步纳入国家发改委编制的《长江中游城市群一体化发展规划》；与新余市、宜春市签订了加快经济转型发展区域合作框架协议；上栗、莲花与湖南浏阳、平江等县市签订了《湘赣边区域开放合作浏阳共识》。

与此同时，政府自身建设不断加强。按照中央要求和省委部署，深入开展第二批党的群众路线教育实践活动，坚决反对“四风”，着力纠治损害群众利益的突出问题，真正让群众受益。办理人大代表议案、建议92件，政协提案130件，办复率100%。建立了监督检查常态化机制，加大了顶风违纪的查处力度。严格落实中央八项规定，严格控制“三公”经费支出，全市“三公”经费支出同比下降25%；开展了打击“两违”、行政执法、超标配备公车、多占办公用房、滥建楼堂馆所、违规参与土地开发和工程建设等专项整治，干部队伍作风进一步好转。进一步加强政风行风建设，对安全生产、环境保护、项目建设中不正确履职或履职不到位情况进行了问责。开展了政府重大投资项目、重大民生工程的跟踪审计，加强了对党政领导干部的经济责任审计。认真履行党风廉政建设责任制，深入推进了党风廉政建设。

各位代表!过去的一年，发展的成绩来之不易。这是省委、省政府和市委正确领导的结果，是市人大、市政协监督支持的结果，是全市上下同舟共济、奋力拼搏的结果。在此，我代表市人民政府，向全市广大工人、农民、知识分子和干部及历任老领导、老同志，向各类企业和纳税人，向各民主党派、工商联、无党派人士和社会团体，向驻萍人民解放军、武警官兵、公安民警，向所有关心、支持、参与萍乡建设和发展的同志们、朋友们和海内外友好人士，表示衷心的感谢和崇高的敬意!

在总结成绩的同时，我们也清醒地看到，我市经济社会发展还存在一些亟待解决的困难和问题：一是经济下行压力加大。重大项目储备不多，部分传统产业的优势逐渐弱化，新的替代产业尚未壮大，安全生产的压力依然存在。二是城乡统筹发展任务繁重。区域发展不均衡，小集镇规划建设整治不到位，功能不完善，农民无序建房问题较为突出。三是生态环境建设仍需加强。部分地区的工矿污染、农业面源污染，水源地保护以及水、大气、土壤等污染治理任务较重。四是公共服务的提供水平与人民群众的期望仍有差距。老百姓的民生保障需进一步加强。五是“为政不为”和“担当不力”现象仍不同程度在一些行政人员当中存在，廉政建设有待加强。对这些困难和问题，我们将高度重视，采取有力措施，切实加以解决。二、奋战2015年：在经济新常态下全力推进转型升级

2015年是全面深化改革的关键之年，是全面推进依法治国的开局之年，也是全面完成“十二五”规划的收官之年，做好今年的工作，责任重大、意义深远。全市务必认真贯彻党的十八大、十八届三中、四中全会和习近平总书记系列重要讲话精神，全面落实省委十三届十次全会及市委十一届九次全会的部署，深入推进“发展升级、小康提速、绿色崛起、实干兴赣”的方针，以稳中求进为工作总基调，主动适应经济发展新常态，坚持转型升级，坚持依法行政，坚持狠抓落实，奋力把经济搞上去，切实把作风正过来，全面开创萍乡经济社会发展各项事业新局面!

2015年全市经济社会发展的主要预期目标是：生产总值增长8.5%，财政总收入增长10%，全社会固定资产投资增长16%，社会消费品零售总额增长12%，城乡居民人均可支配收入分别增长10%和11%，居民消费价格总水平涨幅控制在3%左右，规模以上工业万元增加值能耗下降4%，城镇登记失业率控制在4%以内。

为实现上述目标，主要抓好以下几个方面的工作：

(一)突出转型升级，保持经济稳健增长。力争实现规模以上工业企业增加值450亿元，增长10%；主营业务收入完成1750亿元，增长8%；利税总额完成290亿元，增长8%。

着力主攻工业。大力促进煤炭、陶瓷、水泥、花炮和冶金五大传统产业转型升级。大力发展粉末冶金及先进装备制造、节能环保、新材料、新能源、生物制造和生物农业等战略性新兴产业。利用粉末冶金技术改造提升先进装备制造产业；节能环保产业突出发展环保陶瓷、节能技术产品；新能源产业依托龙头企业形成较完整的产业链；新材料产业依托现有产业基础，加快发展特种材料及应用产品。生物制造和生物农业产业重点发展生物医药、生物制品、绿色生态食品。大力建设以“一区五园”为重点的工业平台。每个工业园重点培育发展1至2个特色产业，力争主导产业的产值和税收贡献达到各自园区的80%以上。以萍乡经济技术开发区和芦溪工业园、莲花工业园为依托，以安源经济转型产业基地、萍乡陶瓷产业基地和上栗经济开发区创建省级工业园区为平台，以设立赣湘开放合作试验区为契机，重点发展特色产业，加快形成各县区以省级工业园区为主，产业基地为辅，特色鲜明、分布合理的产业集群发展体系。加快发展15个产业集群，各产业集群依托重点企业，整合资源，培育3至5个主营业务收入达5亿元以上的龙头企业。

着力抓好项目建设。全年实施重点项目97个，完成投资195亿元。紧紧抓住国家加快推进重大网络、健康养老服务等七大类重大工程建设的有利机遇，谋划和储备一批好项目，努力从享受国家政策渠道上争取更多的资金和项目。重点支持萍矿、中煤科技、萍安钢、华能安源电厂等骨干企业的发展。重点抓好华能二期、比克新能源客车、中材二期、中材风电叶片、方大光伏等重大项目建设。重点跟踪投资5000万元以上的企业。加大对华维电瓷等上市企业的扶持力度。加快推进赣西变压器公司年产5000台干式变压器生产线等一批项目竣工投产。加快推进220KV大仓前和110KV上栗桐木、莲花坊楼输变电重点工程建设。

着力发展民营经济。全面落实国务院和省政府针对中小微企业出台的各项减免税费政策，规范中介机构行为，切实减轻企业负担。消除对民营经济的隐性壁垒，降低投资准入门槛，取消不合理的准入限制，重点引导民营企业进入交通、城市基础设施、教育、医疗、旅游和金融服务等领域，加大对本土中小微企业的支持，放胆、放手、放权鼓励支持民营企业发展，努力形成“重商、爱商、安商”的氛围。

着力发展县域经济。坚持把各具特色的支柱产业作为发展县域经济的支撑，坚持错位和差异化发展，做大做强龙头企业，推动产业集群发展。加快做大县域经济总量，提升发展质量，努力走出一条城乡统筹、产城融合的县域经济发展之路。不断优化城市空间结构，力争将湘东区、上栗县纳入原中央苏区规划范围，莲花县列入中央国家机关及有关单位对口支援范围。

着力推进创新驱动。加快国家创新型试点城市建设步伐。深入推进“质量兴市”和“品牌发展”战略，提升企业产品的核心竞争力。加快市科技创新中心建设，打造一个综合性区域共享服务平台。加快建设粉末冶金产业科技园。加强与高校、科研院所和央企交流合作，提升产业核心竞争力。引进培养一批科技领军人才、科技企业家和创新团队。

(二)推进新型城镇化，努力打造宜居宜业城市。坚持高质量规划、高标准建设、高水平管理，提升城市品位。

统筹城乡规划。按照《萍乡市新型城镇化规划(2014—2020年)》等规划要求，强化规划集中统一。坚持按照三规合一的要求，依法修编全市各项规划，努力做好地下空间的规划。强化规划的刚性约束，严格按照规划建设城镇和乡村。城市规划要注重处理好保护和建设等关系，在保护自然景观的基础上，彰显城市魅力。

加大新型城镇化建设力度。坚持老城区做优、做净、优化基础设施，留住乡愁；新城区建美、建精、建出档次，体现城市特色。注重将老城区改造与萍水河改造统筹推进。逐步将田中湖到双河口之间的沿河路及其周边路网打通，以萍水河为纽带，使萍乡的人脉、文脉、山脉、水脉融为一体，新城区和老城区融为一体。加快推进中环北路、益民路二期、吴楚大道二期、安源大道拓宽改造、垃圾焚烧发电等基础设施项目建设，抓好田中景区、聚龙公园、翠湖公园等园林景观项目建设；着力推进玉湖新区教育园区、城市综合馆、移动大厦、商会大厦、农商大厦、花园小区等新区配套服务设施建设。加快润达国际城市综合体、美佳华商业广场、法莱德国际生活时尚广场建设。打通东门桥头等断头路，加宽光丰铁桥通道。推动萍水南路全面贯通，启动龙古坳、九形岭等旧城改造项目。

加强城市管理。创新城市管理体制和机制，构建网格化、标准化、数字化、精细化管理格局，完善城市基础设施，提高城市管理水平。加快推进铁路、公路沿线环境综合整治，深入推进城市交通治堵工程，优化城市微循环系统，优先发展城市公共交通。强力推进“两违”整治，全面开展治理“超载、道路遗洒”等专项行动，集中整治影响城市环境的顽症问题和关系群众切身利益的突出问题。

构建发达道路交通网。积极协调推进昌栗高速建设，力争2015年底竣工通车。积极推动沪昆高速复线项目落地，力争纳入我省高速公路网规划，并进入国家高速公路网规划。确保319、320国道城区段改造全面完工。积极争取渝长厦快速铁路项目通过我市并设立站点。全力争取萍莲高速纳入全省高速公路建设近期规划并早日开工。完成全市公路升级改造112.58公里。

推进生态文明建设。力争将萍乡列入享受国家资源枯竭城市转型第三阶段(2016-2020年)政策支持名单和国家节能减排财政政策综合示范城市。加快实施全国水生态文明城市建设试点，启动国家森林城市创建工作。完成绿化造林6.93万亩、森林抚育29.3万亩，大力推进低产低效林改造、乡村风景林建设工程，继续实行封山育林，加大矿山环境综合治理力度。积极争取中央和省级农村环保专项资金用于村镇环境综合整治，重点解决农村饮水安全、生活垃圾及生活污水污染等问题，有效改善农村环境。继续通过工程减排、结构减排、管理减排等措施，确保完成主要污染物减排任务。确保化学需氧量、二氧化硫、氨氮和氮氧化物排放量持续下降。加强PM2.5监测，提高空气质量。

(三)发展现代农业，推动农村经济转型。加快构建新型农业经营体系，实现农业总产值和农业增加值均增长2%以上。

稳定农业生产。继续开展粮食稳定增产行动，稳定粮食种植面积，着力提高单产。确保粮食种植总面积120万亩以上，总产量56万吨以上。大力推动农业科技创新，加大对良种繁育、疫病防控、农产品质量安全等关键技术研发和应用的支持力度。

发展现代农业和特色产业。打造一批农业产业化龙头企业，力争市级以上龙头企业达160家。深化农民合作社发展，农民合作社力争达到1820家，创建30个农民合作示范社。着力推进芦溪国家级现代农业示范园区建设。努力做好莲花、上栗、安源等省级现代农业示范园区创建工作。进一步提高农业标准化和机械化水平。培育壮大农业特色优势产业，努力发展农产品精深加工业。争创国家级杂交水稻制种基地，扶持“天涯种业”、“春蕾南方制种公司”打造国家级种子企业。以银河杜仲公司“格林米特杜仲猪”品牌为龙头，推动联户经营，带动全市生猪产业进行标准化养殖、集约化生产。做大做强油菜、油茶产业 侯卫东官场笔记。加快推进319、320国道沿线区域的苗木花卉产业带建设。

加强农业基础设施建设。完善建设用地收储制度，从严打击违法用地和违法建设现象，实现建设占用耕地的占补平衡，确保耕地保有量不减少。优先做好水利项目建设规划，力争碧湖潭水库、萍水河治理等一批重点水源工程进入国家建设盘子。力争新增水利项目总投资10亿元，争取省级以上补助资金6亿元，重点抓好寒山水库、8座病险水闸、7处中小河流治理等新建项目建设。抓好“沃土”工程，完成总投资1.54亿元、14.23万亩的高标准农田建设任务，改造中低产田5万亩，补充新增耕地3800亩。着力推进农村环境整治和美丽乡村建设，不断完善小集镇社会化服务功能配套，加强农村住房建设管理，坚持一户一宅，严格审批程序，切实规范农村个人建房。开展农村人居环境综合整治，全面启动农村生活垃圾和污水处理工作，进一步改善城乡面貌，在推进“物的新农村”建设的同时，切实加强“人的新农村”建设。同时，以最大的决心和最严的举措加强山口岩、锅底潭等水源地保护，确保城乡居民饮水安全。

(四)培育壮大现代服务业，强化转型新支撑。主动适应现代服务业新常态，力争服务业增加值占GDP比重提高1个百分点。

加快发展旅游业。以5A标准加快武功山、安源、杨岐山三大景区建设，按照建设8个以上国家4A级旅游景区要求继续完善景区创建后备库，扎实推进旅游工作六大工程。加快推进智慧景区建设，实现旅游硬件设施、文化项目和服务体系同步全覆盖。围绕核心品牌和特色，突出重点市场的宣传营销，坚持立体式、多渠道创新营销方式。加大旅游招商引资力度，促进旅游区域性综合开发。加快推进“武功山—安源—杨岐山”旅游大环线规划建设，构建便捷通达的景区连通工程，推动全市旅游业蓬勃发展。力争全年接待游客人数和旅游综合收入分别增长25%和35%以上。

加快发展文化产业。积极完善中国安源文化博览园的规划、建设，精心打造文化品牌。重点发展好演艺娱乐、印刷包装、数字动漫和文化创意等一批文化主导产业，努力打造文化创意产业基地。建立健全文化艺术创新机制，繁荣文艺创作。推进基本公共文化服务标准化、均等化建设，完善市、县(区)、乡(镇、街)、村(社区)四级公共文化设施网络。

加快发展电子商贸物流业。加大与大型物流企业对接力度，充分发挥地域和传统产业优势，建设一批大型商品交易市场和特色专业市场。加快推进农村农产品的物流体系和互联网建设。加快永璟、强泰等重点物流项目建设进度，力促上栗县国中安智物流项目成功上市“新三板”。加快推进电子商务推广普及应用，着力打造具有萍乡特色的电子商务产业带，推动商派电子商务产业园和网优电子商务科技园建设，加快中帜汽车汽配城、萍乡先锋电子商务产业园、江西平善堂健康产业交易与服务平台建设。

加快发展金融业。贯彻落实金融改革“1+10”文件。着力做好融资工作，加强金融风险防范。继续做好货币信贷工作，支持各银行业机构围绕重点项目建设、实体经济、现代农业等领域不断创新金融产品，不断巩固市政府与省级银行业机构的合作成果，持续加大有效信贷投放，确保实现“两个不低于”的工作目标。积极开展金融招商，确保交通银行上半年开业。推进小额贷款公司健康发展，健全融资担保体系。抓紧做好企业上市工作，推动一批科技型、成长型的中小企业在股份转让系统挂牌。全力推进信用体系建设。发挥保险“经济助推器”和“社会稳定器”作用，加快发展现代保险服务业。

(五)深化改革扩大开放,激活发展新动力。进一步解放思想,加大改革的力度和开放的广度，不断激发市场活力。

积极稳妥推进重点领域改革。全面做好农村集体土地确权登记颁证工作。大力推进市、县(区)土地流转交易市场建设，适时启动征地制度、农村宅基地制度及农村经营性集体建设用地三项改革。深化财税金融体制改革，着力建立现代财政制度，逐步健全财力与事权匹配的市以下财政体制，适当增强市本级宏观调控能力。大力撬动民间资本，不断创新融资模式，充分发挥BOT(建设-运营-移交)、TOT(移交-经营-移交)、PPP(公私合营或合作)、PFI(私人主动融资)等融资模式的积极作用，化解政府融资难问题。全面落实国资国企改革各项任务，加快促进国有企业建立现代企业制度，积极推进经营性国有资产集中统一监管。全力做好公务用车制度改革，合理有效配置公务用车资源，降低行政成本。

加快简政放权步伐。加快转变政府职能，进一步深化行政审批制度改革，打造好“三单两制一网一评价”。对全市各部门的行政权力和责任进行分类清理，不断完善权力清单、责任清单和负面清单，丰富原有“三单”内涵，进一步优化联合审批流程再造，推行网上联合审批，并将评价纳入行政考核内容。加快推进中介机构改革和发展。取消和下放部分权力，创新和改善政府管理，将政府工作重点转到创造良好发展环境、提供优质公共服务、维护社会公平正义上来。加快市场化改革，公平对待市场主体，让各类市场主体在统一的市场规则中公平竞争。

全面提高开放型经济水平。积极适应全面深化改革的新常态，构建更加开放的平台和市场,大力发展混合所有制经济。加快检验检疫、出口保税区等外向型经济服务平台建设。积极扶持本地进出口企业做大做强，引导有条件的外向型企业走出去。加强招商引资工作，突出产业链招商，重点围绕战略性新兴产业和传统产业，引进一批产业龙头和上下游产业链项目。重点引进一批大企业、大项目，打造重点产业链和产业集群。力争全年引进外资实现增长6%以上，利用省外5000万元以上项目实际进资379亿元。

(六)推进民生改善，开创社会和谐新局面。更加注重改善民生，更加注重繁荣社会事业，努力促进社会和谐进步。

努力促进就业创业。实施就业优先战略和更加积极的就业政策，保持城镇就业率95%以上。鼓励多渠道多形式就业，推动“万众创新，大众创业”，做好以高校毕业生为重点的青年就业工作和农村转移劳动力、城镇困难人员、退役军人就业工作。打造创业服务平台，推动“一体化”创业服务。

不断强化社会保障。坚持全覆盖、保基本、多层次、可持续方针，大力推进“五险”统一征缴，不断扩大社会保险覆盖面。积极稳妥推进保障房建设，抓好棚户区和农村危旧房改造。进一步完善被征地农民基本养老保险政策，全面解决好全市20多万失地农民的养老保险问题。扎实推进扶贫攻坚，落实罗霄山片区区域扶贫攻坚实施规划，大力支持片区区域重点规划项目建设。进一步提高社会救助水平，稳步提高城乡低保、农村“五保”、城镇“三无”、精简退职人员等困难群众保障标准。出台惠民增资政策，提高全社会最低工资标准，加强被雇人员特别是低收入阶层的工资保障。统筹推进养老服务设施建设，支持社会力量发展养老服务。积极落实新型农村合作医疗和城镇居民基本医疗保险政策，扎实推进新型农村社会养老保险，稳步提高社会保障能力和水平。逐步缩小城乡、区域、群体之间的待遇差距，基本建成制度完善、管理科学、覆盖城乡的社会保障体系。

大力发展各项社会事业。推进教育领域综合改革，制定《萍乡市深化教育领域综合改革方案》及相关配套办法。抓好各级各类教育，加快构建现代职业教育体系。进一步做好公立医院改革工作，推进医疗资源有效整合，抓好公共卫生能力项目建设。全力推进国家卫生城市和全国文明城市创建工作。进一步加强食品药品安全监管，确保公众饮食用药安全。着力发展体育事业和体育产业。支持工会、共青团、妇联根据各自章程开展工作，做好国防、人防、民兵预备役等各项工作，争创新一轮全省双拥模范城和全国双拥模范城。积极推进第二次全国地名普查试点工作。切实做好行政区划调整工作。继续抓好计划生育工作，稳定低生育水平。

提高社会治理水平。深入推进基层应急管理规范化建设，进一步提升应急处置水平。继续推行安全管理“三大工程”和“企业可防、政府可控”工程，全面落实新《安全生产法》和安全生产“党政同责、一岗双责”。强化和落实生产经营单位的主体责任，建立生产经营单位负责、职工参与、政府监督、行业自律和社会监督的安全生产格局。推进“本质安全、科学治安”工程。始终保持对非法开采打击的高压态势，加快安全生产管理体制改革，按照“安全生产监管关口前移”的工作思路，把事故苗头和安全隐患消灭在萌芽状态。加强质量和市场监督管理，为人民群众提供安全、可靠的消费环境。及时发现和调处社会矛盾纠纷，提高信访处置能力。深化平安萍乡建设，启动“天网”工程二期建设，进一步提升公众安全感和社会治安好转率。

三、以推进依法行政为重点建设法治政府

紧紧围绕建设法治萍乡的目标，深入推进依法行政，加快建设法治政府。

依法履行政府职能。深入推进政府机构改革，不断完善行政组织和行政程序法律制度，推进机构、职能、权限、程序、责任法定化，推进政府事权规范化、法律化。坚决消除权力设租寻租空间，坚决纠正不作为、乱作为，坚决惩处失职、渎职，切实做到法无授权不可为、法定职责必须为，为经济社会发展创造优良环境。加快建设可持续的基本公共服务制度体系，更好发挥政府提供公共服务、促进社会公平正义的作用。回应社会关切，加强市场监管，切实解决群众反映强烈的安全生产、食品安全、环境保护、社会治安等方面的突出问题，维护经济社会秩序。

健全依法决策机制。对列入重大行政决策范围的事项按规定程序决策。探索制定行政机关重大决策合法性审查办法。建立重大决策终身责任追究制度及责任倒查机制，制定重大决策终身责任追究及责任倒查办法，明确监督主体、监督内容、监督对象、监督程序和方式，实现决策权和决策责任的统一。对决策严重失误或者依法应该及时作出决策但久拖不决造成重大损失、恶劣影响的，严格追究负有责任的领导人员和相关责任人员的法律责任。

着力构建诚信政府。推进行政行为程序规范化，健全行政自由裁量约束机制。完善诚信行政监督制约机制。推动建立行政行为承诺制度，严格履职践诺。推进社会信用体系建设，建设公共信用信息管理系统，引导社会群众诚信守法。推动司法机关建立失信行为监督和惩戒机制，不断增强司法公信力。进一步健全和完善政务信息公开工作体制机制，加大政务公开力度，加强行政服务中心建设，营造优质、高效、便利的政务环境。创新政务公开方式，推行网上电子审批、“一个窗口对外”和“一站式”服务，全面提高政府门户网站公共服务水平。善于使用新媒体手段，进一步发挥政务微博、微信、移动客户端等社交网络和即时通信工具的积极作用，善于用老百姓喜闻乐见的方式，灵活传递政务信息，增强政务公开的影响力和舆论引导力，保障公民的知情权、参与权与监督权。

全面建设廉洁政府。严格落实党风廉政建设责任制，认真履行主体责任和“一岗双责”，不断提升党风廉政建设水平。持之以恒落实中央八项规定，坚决纠正“四风”，严肃财经纪律，强化厉行节约，加强发展环境和机关效能建设，建立健全抓常、抓细、抓长的作风建设机制。加强权力运行监督体系建设，强化制度执行，从严管理干部，把权力关进制度的笼子里。深入开展“为官不为”、“为政不廉”问题专项整治，坚持有腐必惩，有贪必肃，以零容忍态度惩治腐败，着力打造廉洁政府，大力推进风清气正的政治生态建设。

各位代表!雄关漫道真如铁，而今迈步从头越。让我们更加紧密地团结在以习近平同志为总书记的党中央周围，在省委、省政府和市委的正确领导下，解放思想、改革创新，求真务实、团结奋斗，在与全国、全省同步全面建成小康社会的新征程中，为促进萍乡经济社会发展作出新的贡献!
